# Stavros Niarchos Park Walk for people with dementia: Memory and Senses

**DOI:** 10.1002/alz.090658

**Published:** 2025-01-09

**Authors:** Patra Blekou, Panagiota Zoi, Antigoni Athina Leonti, Meropi Kalimniou, Paraskevi Sakka

**Affiliations:** ^1^ Athens Alzheimer Association, Athens, Attica Greece

## Abstract

There are 160.000 people living with dementia and 280.000 with Mild Cognitive Impairment (MCI) in Greece. Accordingly, more than 1.500.000 family carers are involved their care. COVID‐19 pandemic and social distancing measures caused significant decline, both in an overall aspect of the people with dementia and their carers, and in specific domains (mostly communication, mood and socialization).

Rapidly aging global population increases the demand for programs activating elderly people in general and people with cognitive disorders in particular. Museums and cultural centers are well placed to provide such interventions. Many studies indicate that activities such as visiting museums and cultural centers, engaging in visual and performing arts, enjoying music, dancing etc., have positive impact on people with dementia and lead to alleviation of depression symptoms and improvement of cognitive functions.

In order to dismantle barriers and provide equal opportunities to people with dementia in engaging in cultural activities in Greece, Alzheimer Athens with the support of the Stavros Niarchos Foundation Cultural Center (SNFCC) has designed a guided walking tour in the Stavros Niarchos Park, where the largest public Mediterranean garden in the world is located. On a monthly basis, during an 1,5 hour tour, people with MCI and dementia are acquainted with a rich variety of flora, trees, shrubs, as well as an extensive selection of indigenous Greek aromatic plants accompanied by two health professionals from Alzheimer Athens. Later on, they visit a temporary art exhibition located at the SNFCC Esplanade. The walk ends with specially designed dual task activities using the information provided in the tour. With an average of 25 participants per tour, more than 300 people with cognitive deficits have participated since January 2023.

Overall, this intervention aims to combine physical and mental activation using the senses (touch, smell, sight and hearing), to improve mood and to promote socialization of elderly participants through an interactive, educational and relaxing journey.

